# Interrelations of Synthesis Method, Polyethylene Glycol Coating, Physico-Chemical Characteristics, and Antimicrobial Activity of Silver Nanoparticles

**DOI:** 10.3390/nano10122475

**Published:** 2020-12-10

**Authors:** Amirah Shafilla Mohamad Kasim, Arbakariya Bin Ariff, Rosfarizan Mohamad, Fadzlie Wong Faizal Wong

**Affiliations:** 1 Department of Bioprocess Technology, Faculty of Biotechnology and Biomolecular Sciences, University Putra Malaysia, Serdang 43400, Selangor, Malaysia; mirafilla123@gmail.com (A.S.M.K.); arbarif@upm.edu.my (A.B.A.); farizan@upm.edu.my (R.M.); 2 Bioprocessing and Biomanufacturing Research Centre, Faculty of Biotechnology and Biomolecular Sciences, University Putra Malaysia, Serdang 43400, Selangor, Malaysia

**Keywords:** chemical synthesis, biological synthesis, silver nanoparticles, polyethylene glycol, antimicrobial activity

## Abstract

Silver nanoparticles (AgNPs) have been found to have extensive biomedical and biological applications. They can be synthesised using chemical and biological methods, and coated by polymer to enhance their stability. Hence, the changes in the physico-chemical characteristics of AgNPs must be scrutinised due to their importance for biological activity. The UV-Visible absorption spectra of polyethylene glycol (PEG) -coated AgNPs displayed a distinctive narrow peak compared to uncoated AgNPs. In addition, High-Resolution Transmission Electron Microscopy analysis revealed that the shapes of all AgNPs, were predominantly spherical, triangular, and rod-shaped. Fourier-Transform Infrared Spectroscopy analysis further confirmed the role of PEG molecules in the reduction and stabilisation of the AgNPs. Moreover, dynamic light scattering analysis also revealed that the polydispersity index values of PEG-coated AgNPs were lower than the uncoated AgNPs, implying a more uniform size distribution. Furthermore, the uncoated and PEG-coated biologically synthesised AgNPs demonstrated antagonisms activities towards tested pathogenic bacteria, whereas no antagonism activity was detected for the chemically synthesised AgNPs. Overall, generalisation on the interrelations of synthesis methods, PEG coating, characteristics, and antimicrobial activity of AgNPs were established in this study.

## 1. Introduction

Nanoparticles (NPs) can be defined differently, depending on the types of materials, fields, and applications [1]. However, the particles in the size range from 1 nm to 100 nm are generally considered to be NPs [2]. Additionally, NPs have different physical and chemical properties from their bulk materials; these properties are affording the exploitation of NPs for various applications [3]. These NPs can also be classified into organic (e.g., carbon nanotubes, fullerenes, and chitosan) and inorganic (e.g., zinc oxide, gold, iron, and cadmium sulphide) groups [4].

Silver nanoparticles (AgNPs) are one of the important metallic nanoparticles that have received great attention because of their usage in biomedical and biological applications, particularly as an antimicrobial agent [5]. AgNPs have been intensely studied due to their intrinsic unique properties (i.e., optical behaviour, conductivity, chemical stability, and catalytic activity) [6]. Furthermore, AgNPs can be synthesised using chemical and biological methods. The production of AgNPs using chemical synthesis can be realised using several methods, including chemical reduction, thermal decomposition, electrochemical, laser ablation sputtering, and photoreduction [7,8]. The chemical reduction and electrochemical pathways are the most common scheme for the synthesis of NPs [5,9]. The reducing agents involved in the synthesis of NPs could be either organic or inorganic compound (e.g., sodium borohydride (NaBH_4_), sodium citrate, ascorbate, and polyethylene glycol (PEG)) in order to reduce particular ions in aqueous or non-aqueous solutions [5]. However, the silver dispersions obtained from some of the methods are only stable at relatively low concentrations of metal; thus, these methods are deemed not suitable for upscale production, and are also high-cost [7]. Among the methods used, chemical reduction of silver salt with or without a stabilising agent (e.g., polymer and surfactant), appears to be the most common method to synthesise AgNPs [8]. Sondi et al. [7] reported that the chemical reduction of silver nitrate with the presence of a stabilising agent, Daxad 19, in aqueous solution, obtained highly concentrated and stable dispersion of NPs.

AgNPs can also be prepared using biological sources (e.g., plant extract, bacteria, and fungi); this approach is therefore more environmentally-friendly and cost-effective [10]. However, plant extract-based synthesis appears to be the most appealing approach due to the easier subsequent extraction process compared to microbial routes, which require aseptic conditions for cultivation and laborious work in maintaining the cells [11]. In addition, the usage of plant extract may also offer many benefits, including high source availability, eco-friendliness, safety to handle, and containing a wide range of plant metabolites [12]. Basically, *Aloe vera* extract contains various components (i.e., vitamins, enzymes, sugars, salicylic acids, and amino acids), and the benefits of these natural components in the biomedical field, including anti-inflammatory, anti-arthritic activity, and antibacterial effects, have been well-reported [13,14,15,16]. These active components can act as electron shuttles in metal reduction; and some constituents are responsible for the formation of AgNPs [16].

The biggest challenge associated with AgNPs synthesis is their instability and susceptibility to agglomeration intrinsic characteristics, which are resulting in the formation of larger-size AgNPs [3]. Improving the stability of AgNPs can be achieved through steric repulsion or electrostatic repulsion effects, which can be aided by polymers such as polyethylene glycols (PEGs), polyvinyl alcohols (PVAs), and polyvynil pyrolidons (PVP), and surfactant molecules [17]. Electrostatic repulsion effects are promoted when the ionic surfactant molecules (e.g., sodium dodecyl sulfate (SDS) and cetyltrimethylammonium bromide (CTAB)) enhanceg the surface charge of the dispersed phase, providing electrostatic protection on the NPs to adhere to one another [17]. Additionally, Sarkar et al. [18] highlighted that polymers (e.g., polysaccharides, polyacrylamide, and PEG) and ligands (e.g., citrates, amines, peptides, and lipids) are widely used as capping agents for the surface modification because these substances can control the rate of reduction of metal ions and the aggregation process of the metal clusters.

Over the past decades, many researchers have worked on the synthesis of AgNPs and their applications as antimicrobial agents in their original form [19]. Nevertheless, comprehensive studies on the synthesis, polymer coating, characterisation, and biological application of AgNPs remain scarce. Hence, the primary aim of this study is to investigate the interrelations of synthesis methods (chemical and biological syntheses), PEG coating, physico-chemical characteristics (in terms of optical properties, molecular components and structure, stability, morphology, surface charge, and polydispersity), and biological activities (antimicrobial) of uncoated and PEG coated AgNPs. The findings present a profound implication on the future development of synthesis and coating methods of AgNPs, with respect to desired characteristics for a particular application.

## 2. Materials and Methods

### 2.1. Sample Preparation

#### 2.1.1. Chemical Synthesis of AgNPs

The chemical synthesis of the AgNPs was carried out based on the method of Guarrotxena and Braun [20] with slight modification. A mass of 90 mg of silver nitrate (AgNO_3_, Sigma-Aldrich, Gillingham, UK) was added to 500 mL of distilled water and heated until boiling. Then, 10 mL of a 1% sodium citrate (Sigma-Aldrich, Gillingham, UK) solution was added to the mixture, followed by continuous boiling for 15 min. The mixture was subsequently centrifuged for 30 min at 1055× *g* (3135 rpm) to precipitate large NPs aggregates, that could have been formed during the synthesis. The supernatant containing AgNPs was then collected for further characterisations.

#### 2.1.2. *Aloe vera* Extract Preparation

The extraction of Aloe vera was carried out based on the method of Chandran et al. [15] with slight modification. A mass of 30 g of Aloe vera leaves were washed, finely cut, and boiled in 100 mL of distilled water for 15 min. The extraction product was used for subsequent experiment, and the remaining of Aloe vera extract was stored at 4 °C.

#### 2.1.3. Biological Synthesis of AgNPs Using Aloe vera Extract

The biological synthesis of the AgNPs was carried out based on the method of Chandran et al. [15] with slight modification. A volume of 5 mL of 0.01 M AgNO_3_ solution was added with 2.5 mL of 30% ammonia solution. Then, 5 mL of the Aloe vera extract was added into the mixture. The final volume of the mixture was made up to 50 mL with distilled water and then incubated at room temperature. The appearance of a faint yellow colour after 24 h indicated the formation of AgNPs. The mixture was subsequently centrifuged for 30 min at 1055× *g* (3135 rpm) to precipitate large NPs aggregates and remove residuals of the Aloe vera extract that could have been formed during the synthesis. The supernatant containing AgNPs was then collected for further characterisations.

#### 2.1.4. Surface Coating of AgNPs with Polyethylene Glycol (PEG)

The PEG coating of the AgNPs was carried out based on the method of Nabiyouni et al. [21] with slight modification. A mass of 10 g of PEG (Sigma-Aldrich, Gillingham, UK), with a molecular weight of 8000 g/mol, was dissolved in 90 mL of distilled water and stirred using a magnetic stirrer at medium speed for 24 h and room temperature. Then, the PEG solution was mixed with a 50 mL of AgNPs solution and stirred using a magnetic stirrer at medium speed and room temperature for 48 h.

### 2.2. Characterisations of AgNPs

#### 2.2.1. UV-Visible Spectrophotometry Analysis

The optical properties of the AgNPs obtained after the synthesis and PEG coating process were investigated using UV-Visible spectrophotometer (UvLine 9400, Secomam, Alès, France). In brief, the samples were filled into a cuvette and placed in a measurement chamber, and then scanned over the wavelength range from 300 nm to 600 nm at room temperature. The spectra of the samples were then presented in the graph of absorbance as a function of wavelength (nm).

#### 2.2.2. High Resolution—Transmission Electron Microscopy (HR-TEM) Analysis

The size and morphology of the AgNPs were analysed using high resolution transmission electron microscopy (HR-TEM) (HITACHI H-700, Tokyo, Japan). The morphological analysis of the AgNPs was carried out based on the method of Kathiraven et al. [22] with slight modification. The images were obtained with acceleration of 80 kV at room temperature. In brief, a drop of the AgNPs sample was placed on a copper grid coated with carbon film, and allowed to dry at room temperature prior to viewing. The dimension and size distribution of the samples were measured using the ImageJ version 1.52a software (NIH).

#### 2.2.3. Fourier-Transform Infrared Spectroscopy (FTIR) Analysis

The presence of different functional groups on the AgNPs was determined using Fourier-Transform Infrared Spectroscopy (FTIR) Nicolet Nexus 470 (Thermo Fisher Scientific, Madison, WI, USA) with a resolution of 4.0 cm^−1^ at ambient temperature. The analysis was carried out based on the method of Awwad et al. [11] with slight modification. A potassium bromide (KBr) disc containing 1.0 mg of the sample and 0.1 mg of fine grade KBr were used at a wavenumber range from 400 to 4000 cm^−1^.

#### 2.2.4. Dynamic Light Scattering (DLS) and Zeta Potential (ZP) Measurements

The hydrodynamic sizes of samples were analysed by dynamic light scattering (DLS) using Zetasizer Nano (Malvern Instruments, Malvern, UK). The analysis was carried out based on the method of Marsalek [23] with slight modification. At least five consecutive measurements were recorded and averaged to calculate the average size. The parameters (absorption and refractive index) that were used for the synthesised AgNPs were: Absorption of 0.1, refractive index of 2.0; while the parameters (viscosity and refractive index) used for dispersants (distilled water) were: Viscosity of 1.0031 cP, refractive index of 1.33. Three independent replicates of samples (approximately 3 mL) were filled into a cuvette with zero cross-flow. Then, the particles’ electrostatic charges (zeta potentials) were also evaluated, using the laser doppler electrophoresis technique, where a volume of approximately 1.6 mL of the samples was injected into the Zetasizer Nano instrument’s cuvette (Malvern Instruments, Malvern, UK).

#### 2.2.5. AgNPs’ Antimicrobial Activity Evaluation

The evaluation of the AgNPs’ antimicrobial activity was carried out based on the method of Mohd Yusof et al. [24] with slight modification. Agar well-diffusion method against Gram-positive (*Staphylococcus aureus, Staphylococcus epidermidis*) and Gram-negative (*Escherichia coli* and *Salmonella* sp.) bacteria was used. The bacteria were grown in nutrient broth and standardised to 0.5 McFarland (turbidity) (approximately 1.5 × 10^8^ colony-forming units per mL). Ampicillin was used as positive control, while distilled was used as negative control. The synthesised AgNPs were subjected to freeze-drying to obtain a dry powder form for antimicrobial assessment. Simultaneously, the agar was punched with a sterile borer to create a sample well, and about 100 µL of samples with a concentration of 100 µg mL^−1^ were deposited into each well. The plate was incubated at 37 °C overnight, and the diameter of the inhibition zone formed was then measured.

### 2.3. Statistical Analysis

All data were analysed using Microsoft Office Excel 2016 (Microsoft Corporation, Redmond, WA, USA). Data were presented as mean ± standard deviation (S.D). Statistical analysis was also performed using one-way analysis of variance (ANOVA) with Microsoft Excel 2016 using default parameters, which were considered significant at *p* < 0.05. ImageJ version 1.52a software (NIH) was used for the size distribution analysis, and the data were presented as mean ± standard deviation (S.D) by counting 100 particles.

## 3. Results

### 3.1. UV-Visible Absorption Spectrophotometry Analysis

**Figure 1:**
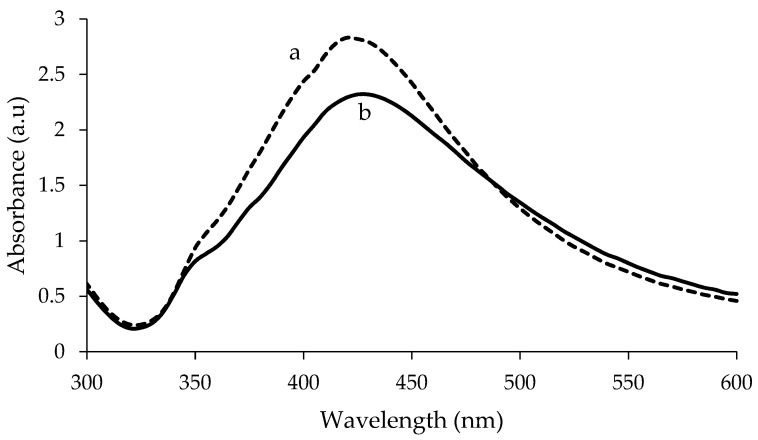
Comparison of UV-Visible spectrum between polyethylene glycol (PEG)-coated (**a**) and uncoated (**b**) chemically synthesised Silver nanoparticles (AgNPs).

**Figure 2:**
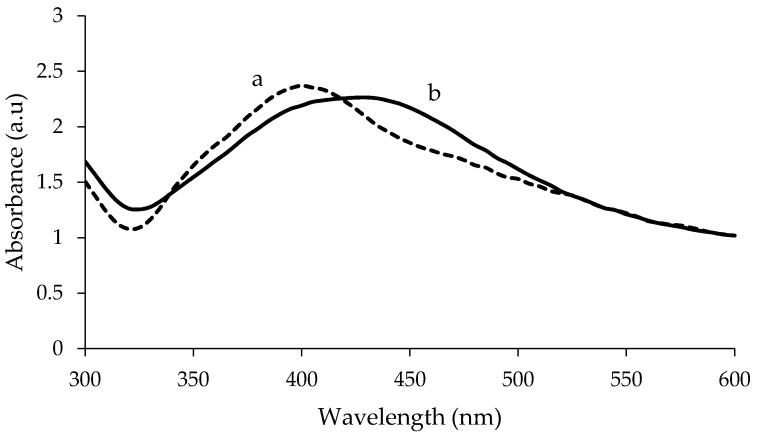
Comparison of UV-Visible spectrum between PEG-coated (**a**) and uncoated (**b**) biologically synthesised AgNPs.

The visible spectra of uncoated and PEG-coated AgNPs from chemical and biological syntheses are shown in Figure 1 and Figure 2, respectively. The analysis was done for the samples with a reaction time of 24 h. Distilled water was used as reference before the spectra of the synthesised AgNPs were recorded. The spectra of the synthesised AgNPs were recorded in the range of wavelength from 300–600 nm. For chemically synthesised AgNPs, narrow peaks can be observed at 425 nm and 420 nm, for uncoated and PEG-coated AgNPs, respectively (Figure 1). Whereas, for biologically synthesised AgNPs, broad peaks can be observed at 430 nm and 400 nm, for uncoated and PEG-coated AgNPs, respectively.

The wavelength and absorbance values of the absorption peaks for the uncoated AgNPs from chemical and biological synthesis were found to change after being coated with PEG (Figure 1 and Figure 2). In addition, the absorbance values of the peaks for the uncoated AgNPs obtained from both synthesis methods were found to increase after the PEG coating. For chemically synthesised AgNPs, the absorbance value of the peak increased from 2.320 to 2.832 following the PEG coating. Whereas for PEG-coated biologically synthesised AgNPs, the peak absorbance value increased from 2.264 to 2.371.

### 3.2. High Resolution Transmission Electron Microscopy (HR-TEM) Analysis

**Figure 3:**
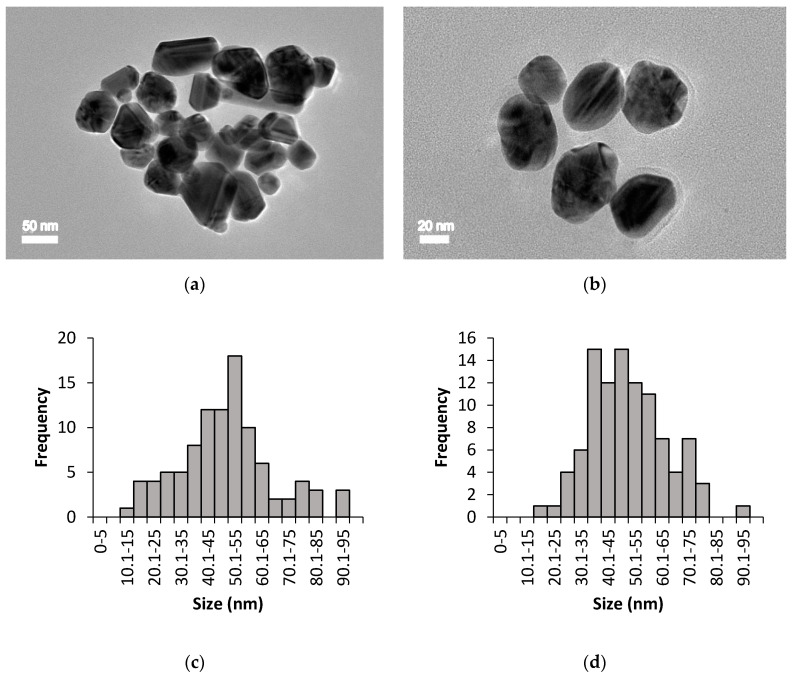
HR-TEM imaging for chemically synthesised AgNPs: (**a**) Uncoated AgNPs, (**b**) PEG-coated AgNPs, (**c**) size distribution of uncoated AgNPs with an average size of 49.26 ± 13.76 nm, and (**d**) size distribution of PEG-coated AgNPs with an average size of 50.43 ± 19.10 nm.

**Figure 4:**
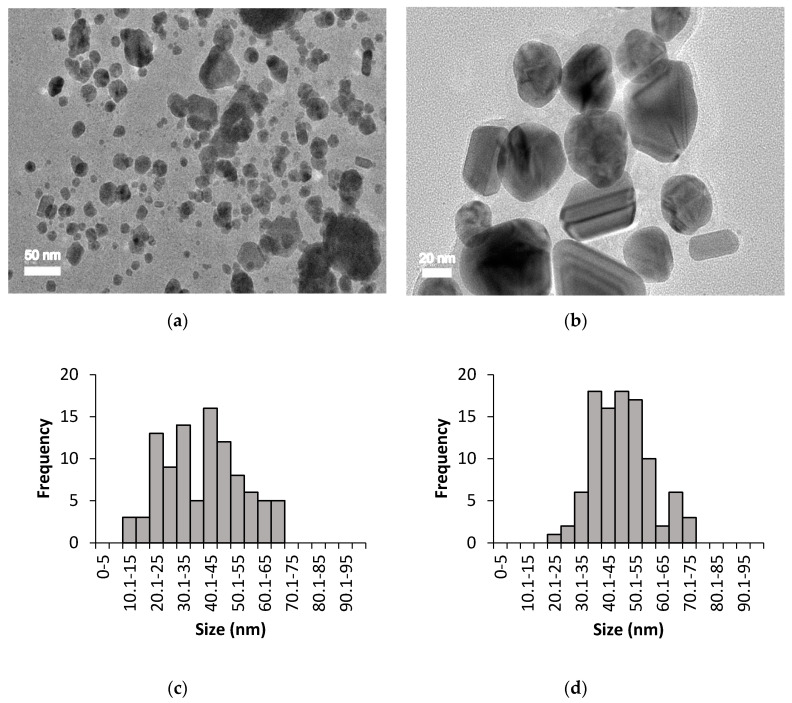
HR-TEM imaging for biologically synthesised AgNPs: (**a**) Uncoated AgNPs, (**b**) PEG-coated AgNPs, (**c**) size distribution of uncoated AgNPs with an average size of 40.26 ± 14.27 nm, and (**d**) size distribution of PEG-coated AgNPs with an average size of 47.36 ± 10.61 nm.

The size, shape, and distribution of chemically and biologically synthesised AgNPs were evaluated through HR-TEM observation. Figure 3 and Figure 4 show the HR-TEM images and size distribution of uncoated and PEG-coated AgNPs (prepared after 24 h of incubation time) from chemical and biological syntheses, respectively. The results clearly show that the sizes and shapes of the nanoparticles were considerably developed after 24 h of reaction time. For chemically synthesised AgNPs, the average sizes of the uncoated and PEG-coated AgNPs were 49.26 ± 13.76 nm and 50.43 ± 19.10 nm, respectively (Figure 3c,d). Meanwhile, for biologically synthesised AgNPs, the average sizes of the uncoated and PEG-coated AgNPs were 40.26 ± 14.27 nm and 47.36 ± 10.61 nm, respectively (Figure 4c,d).

The size distribution was determined using ImageJ software, which was based on 100 particles analysis (sample from 24 h PEGylation reaction). The average sizes of the AgNPs obtained from both synthesis methods were found to be below than 100 nm (Figure 3 and Figure 4), which were in accordance with the previous report [25]. The trends of the graphs show slight increments in the sizes of the PEG-coated AgNPs compared to the uncoated AgNPs (Figure 3c,d and Figure 4c,d), which can be attributed to the presence of additional PEG layer that coated the surface of the AgNPs [3]. Based on the analysis of all samples, mixtures of spherical, triangular, and rod shape AgNPs were observed (Figure 3 and Figure 4).

### 3.3. Fourier-Transform Infrared Spectroscopy (FTIR) Analysis

**Figure 5:**
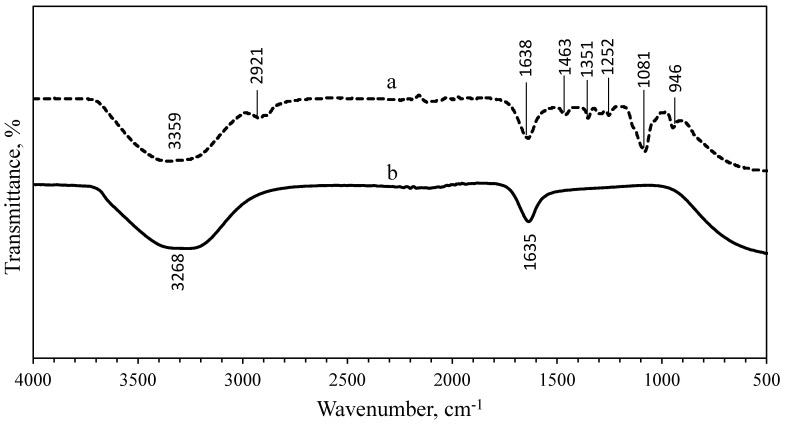
Comparison of FTIR spectrum between PEG-coated (**a**) and uncoated (**b**) chemically synthesised AgNPs.

**Figure 6:**
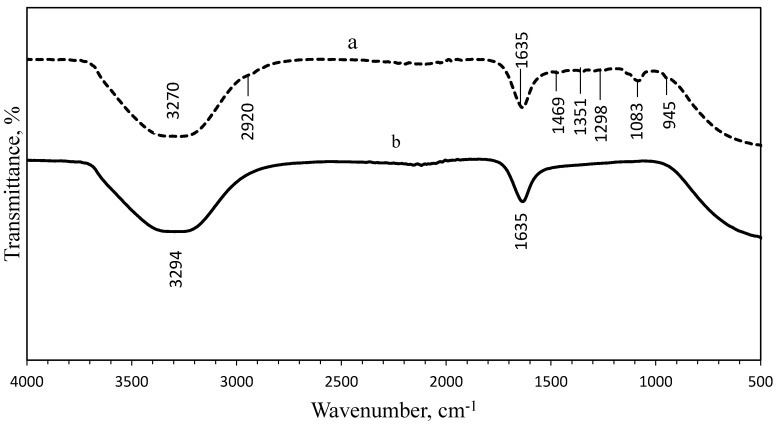
Comparison of FTIR spectrum between PEG-coated (**a**) and uncoated (**b**) biologically synthesised AgNPs.

FTIR analysis was performed to identify the presence of organic materials and other functional groups, that are potentially bound on the surface of the NPs. The spectra of the uncoated and PEG-coated AgNPs obtained from both chemical and biological syntheses were compared (Figure 5 and Figure 6). Figure 5 shows the spectrum of uncoated AgNPs (chemically synthesised), wherein two intense peaks can be identified at 3268 cm^−1^ and 1635 cm^−1^ wavenumbers; while for the PEG-coated AgNPs, multiple peaks were observed at multiple wavenumbers: 3359 cm^−1^, 2921 cm^−1^, 1638 cm^−1^, 1463 cm^−1^, 1351 cm^−1^, 1252 cm^−1^, 1081 cm^−1^, and 946 cm^−1^. For biologically synthesised AgNPs, two peaks were observed at 3294 cm^−1^ and 1635 cm^−1^ wavenumbers for the spectrum of uncoated AgNPs; while for the PEG-coated AgNPs, multiple peaks were detected at 3270 cm^−1^, 2920 cm^−1^, 1635 cm^−1^, 1469 cm^−1^, 1351 cm^−1^, 1298 cm^−1^, 1083 cm^−1^, and 945 cm^−1^ wavenumbers (Figure 6).

The wide spectrum peaks detected at 3268 cm^−1^ and 3359 cm^−1^ on Figure 5; 3294 cm^−1^ and 3270 cm^−1^ on Figure 6, correspond to strong stretching vibrations of hydroxyl functional groups, due to the non-reactivities of some hydroxyl groups during the oxidation and also absorption of moistures on the highly reactive surfaces of NPs [9,26]. Table 1 shows the absorption peaks and vibrational assignments with functional groups interpretations of synthesised AgNPs.

### 3.4. Dynamic Light Scattering (DLS) and Zeta Potential (ZP) Measurements

The polydispersity indexes (PDIs) for uncoated and PEG-coated chemically synthesised AgNPs were 0.514 and 0.476, respectively (Table 2). Meanwhile, the PDIs for uncoated and PEG-coated biologically synthesised AgNPs were 0.571 and 0.534, respectively. Based on the results from UV-Visible absorption, the biologically synthesised AgNPs recorded a broader surface plasmon resonance (SPR) peak compared to the chemically synthesised AgNPs, which indicated a broader size distribution. The phenomenon was further confirmed by this DLS analysis, as indicated by the higher polydispersity index (PdI) value obtained for the biologically synthesised AgNPs (0.571) compared to chemically synthesised AgNPs (0.514).

The ZP values of the AgNPs from both synthesis methods, for uncoated and PEG-coated, are also shown in Table 2. Generally, there are several ranges of ZP values which indicate the stability of AgNPs. Values ranges of ±0–10 mV, ±10–20 mV, ±20–30 mV, and >±30 mV represent the highly unstable, relatively unstable, moderately stable, and highly stable colloid, respectively [3]. The ZP values for the uncoated AgNPs (chemically synthesised), PEG-coated AgNPs (chemically synthesised), and PEG-coated AgNPs (biologically synthesised) were −32.3 mV, −36.4 mV, and −30.6 mV, respectively; whereas the uncoated AgNPs from biological synthesis recorded a value of 29.8 mV.

Hence, only the uncoated AgNPs (from biological synthesis) have a positively charged ZP on their surfaces, which might be due to the presence of ammonia solution during the synthesis process. Ammonia was used to facilitate the formation of a soluble silver complex (diamine silver (Ⅰ) chloride) for the reduction to take place in order to synthesise AgNPs [15]. Meanwhile, the PEG-coated AgNPs from biological synthesis possessed negatively charged ZP, due to the presence of the PEG coating layer.

### 3.5. Antimicrobial Tests of AgNPs

**Figure 7:**
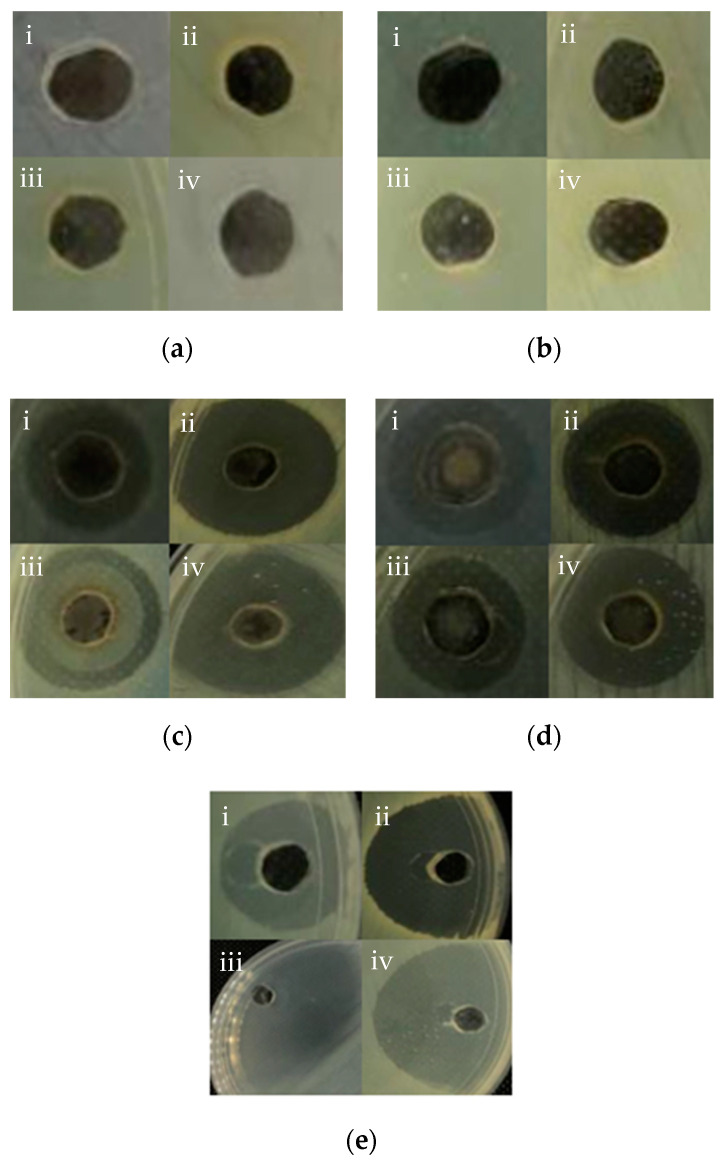
Inhibition zones of 100 µL of different AgNPs samples; (**a**) uncoated AgNPs from chemical synthesis, (**b**) AgNPs from chemical synthesis, with PEG-coating, (**c**) uncoated AgNPs from biological synthesis, (**d**) AgNPs from biological synthesis, with PEG-coating, and (**e**) Ampicillin (control) against pathogenic bacterial strains (i) *Escherichia coli*, (ii) *Staphylococcus aureus*, (iii) *Salmonella* sp., and (iv) *Staphylococcus epidermidis*.

All of the synthesised AgNPs demonstrated bactericidal activities against all tested pathogenic bacteria except for the chemically synthesised AgNPs (for uncoated and PEG-coated), as no inhibitory zone was evident (Figure 7 and Table 3). The antagonistic properties of the uncoated and PEG-coated AgNPs from biological synthesis exhibited high antagonism against *Salmonella* sp., with inhibitory zones of 26.00 mm and 18.00 mm, respectively. Meanwhile, the least antagonistic activity was detected against *E. coli* for uncoated and PEG-coated AgNPs, with inhibitory zones of 13.00 mm and 12.00 mm, respectively. Notably, the inhibitory zones of the PEG-coated AgNPs were found to be smaller (about 7.7–31%) compared to the uncoated AgNPs (Table 3). This can be explained by the presence of the PEG layer on the surfaces of the NPs, which reduced the AgNPs’ chemical reactivities, and hence their antimicrobial activities [28].

## 4. Discussion

In this study, AgNPs were synthesised using chemical and biological methods. The AgNPs were then characterised using UV-Visible spectrophotometry, HR-TEM, FTIR, and DLS. Biological synthesis offers advantages over the chemical synthesis in terms of sustainability, eco-friendliness, and production cost. The synthesised AgNPs were subsequently coated with PEG, and their characteristics were compared to uncoated AgNPs. The PEG-coated AgNPs from both synthesis methods exhibited a higher degree of stability compared to the uncoated AgNPs based on the UV-Visible adsorption, HR-TEM, and DLS analyses. Based on the UV-Visible absorption spectra analysis, the absorption peaks of AgNPs were detected at a wavelength ranging from 412 to 437 nm, which are typical for AgNPs; thus confirming the formation of AgNPs [26]. Besides, previous researchers suggested that the size, shape, and dispersion of NPs also have a direct effect on the spectrum of NPs [6]. When the surfaces of AgNPs are coated with a PEG layer, diffusion barriers are created. Additionally, some of irregular-shaped AgNPs are changed to spherical shape, increasing their stability and preventing agglomeration [29].

Meanwhile, the recorded wavelengths of the absorption peaks were still found to be falling under the range of typical AgNP’s wavelength; therefore, these results proved that there is no significant change in the value of the SPR after the AgNPs were coated with PEG [3]. Basically, SPR is defined as the phenomenon that occurred at the surface of metal NPs when an incident light beam hit the surface at a particular angle; hence, SPR phenomenon also can be monitored using a UV-Visible spectrophotometer [30]. The changes in the wavelength of the absorption peaks were a blue shift, which mean a change towards a shorter wavelength due to the shape of AgNPs. The shape of AgNPs could be more spherical, when the wavelength of the absorption peaks is shorter; hence, the longer the wavelength of the adsorption peaks, the higher the variation of the AgNPs shape obtained [31]. The change in absorbance for the PEG-coated AgNPs occurred due to the addition of PEG polymer; the presence of additional layer of PEG (coating) and the ongoing reduction of silver ions to silver atoms, resulted in the increment of the AgNPs’ size and number formed [26].

The HR-TEM images elucidated the formation of AgNPs, which can be correlated with the spectrum of SPR band in the UV-Vis absorption determined earlier. The uncoated AgNPs from biological synthesis were found to be agglomerated, which might be due to the absence of stabilising or coating agents during the synthesis. The lower degree of agglomeration for the uncoated AgNPs from chemical synthesis was probably due to the presence of sodium citrate, which acted as a stabilising agent [32]. Generally, the shape of AgNPs can be modified with the addition of different concentrations of reducing agents. Chandran et al. [15] reported that triangular-shaped AgNPs were obtained when a lower volume of Aloe vera extract (around 0.5 mL) was used, while spherical AgNPs were becoming prevalent as the Aloe vera volume was increased to more than 5 mL.

Meanwhile, the FTIR results showed that the bands at 2921.16 cm^−1^ and 2920.02 cm^−1^ wavenumbers were attributed to the aliphatic C-H stretching of aldehyde groups because some solutions failed to oxidise to carboxylic acid [9,26]. Therefore, the peaks at 2921.16 cm^−1^, 2920.02 cm^−1^, 946.58 cm^−1^, and 945.17 cm^−1^ wavenumbers can be assigned for −CH_2_ stretching vibration and −CH out-of-plane bending vibration, which confirmed the presence of PEG on the surface of the NPs [27]. The bands at 1635.16 cm^−1^, 1638.96 cm^−1^, 1635.09 cm^−1^, and 1635.33 cm^−1^ wavenumbers were related to the carbonyl (C=O) and N-H stretching vibrations in amide linkages and formed between the PEG and AgNPs, while the peaks at 1463.94 cm^−1^ and 1469.17 cm^−1^ wavenumbers corresponded to aromatic compounds [27]. The peaks at 1351.29 cm^−1^ and 1351.61 cm^−1^ wavenumbers were corresponding to the combination band of O–C–H, while the peaks at 1081.14 cm^−1^ and 1083.22 cm^−1^ wavenumbers were corresponding to hydroxyl groups, indicating the functionalisation of the NPs with PEG molecules [9,27]. The comparison of spectra between the uncoated and PEG-coated AgNPs demonstrated slight reductions and shifts in several peaks’ intensities and positions, as a result of the coating.

In addition, the PDIs indicated the dispersity degrees of AgNPs: a value range of <0.1, 0.1–0.4, and >0.4 represents the highly monodisperse, moderately disperse, and highly polydisperse distributions, respectively [3]. These results implied that all of the samples were moderately dispersed even after being coated with PEG; however, the values were found to decrease compared with the uncoated AgNPs. Further, the negative charges of the uncoated AgNPs (from chemical synthesis) were ascribed to the presence of sodium citrate during the synthesis process, wherein the absorption of residual citrate ions on the AgNPs during the synthesis may have taken place [33]. The elevated absolute ZP values indicated the presence of high electric charges, which were more than ±30 mV, pointing to the presence of huge repellent forces in the particles, resulting in the stabilisation of the NPs in the medium [3,34]. Thus, the particles did not undergo coalescence, and therefore no aggregation occurred, which promoted long term stability of the particles.

Then, the PEG-coated and uncoated AgNPs were subjected to antimicrobial tests against pathogenic bacteria. Biologically synthesised AgNPs, which exhibited higher antimicrobial potency (as highlighted in present study) and biocompatibility, can be exploited for several biomedical applications, including the development of biomedical devices and consumer products. Although PEG-coated AgNPs exhibited lesser antimicrobial effects compared to the uncoated AgNPs, their usage is generally preferred, owing to their minimal non-specific interactions with other proteins, and higher capacity for control release of silver ions from the AgNPs.

In addition, the toxicities of AgNPs towards bacterial strains are dependent upon the direct interaction between the AgNPs and the targeted pathogens; therefore, the chemical surface properties of AgNPs are important [28]. Furthermore, the antimicrobial properties of AgNPs may also depend on the size of AgNPs, wherein smaller sized AgNPs generally exhibiting greater antimicrobial activity due to their larger surface area to volume ratio [35,36]. Nevertheless, PEG-coated AgNPs are much more preferable for biomedical and biological applications because the coated layers will enhance the receptor-mediated delivery of the nanoparticles to targeted cells and minimise the non-specific interactions with other proteins [37]. Mechanistically, silver cations released from AgNPs can attach to the bacterial cell membrane through electrostatic forces, and these interactions may in turn distort the membrane structure and damage the cell wall, resulting in the leakage of intracellular components and end with cell death [10]. The AgNPs’ antimicrobial properties are also dependent on their size, environmental conditions (i.e., size, pH, and ionic strength), and capping agent [5].

Meanwhile, the negative results obtained for chemically synthesised AgNPs (both uncoated and PEG-coated) might be due to the oxidation of the AgNPs and their inability to release the silver ions to destroy the bacteria. Prema [38] highlighted that NPs require sufficient incubation time during the antimicrobial activity evaluation to visualise the inhibition zone. Additionally, it was reported previously that biologically synthesised AgNPs were able to produce inhibition zones in agar well diffusion assay at low concentration compared to chemically synthesised AgNPs [39]. Additionally, the size and capping agent of AgNPs may affect the antimicrobial properties of AgNPs towards the bacteria [5].

## 5. Conclusions

The formation of AgNPs was initially confirmed by UV-Visible spectrophotometry analysis, wherein multiple discernible peaks at 400–430 nm were observed. The morphological study of the synthesised AgNPs using HR-TEM revealed that the predominant shape was spherical (86.86%), followed by triangular (8.00%) and rod (5.14%).

Additionally, FTIR spectra analyses confirmed that the biomolecules in Aloe vera leaves extract were responsible for reducing and capping of the AgNPs. The PEG-coated AgNPs exhibited better size distributions, as evidenced by the smaller values of PDI compared to uncoated AgNPs. Additionally, the ZP values indicated that the samples of chemically synthesised (uncoated and PEG-coated) and PEG-coated biologically synthesised AgNPs were highly stable colloids, while the uncoated biologically synthesised AgNPs were moderately stable. Overall, the characteristics of the PEG-coated AgNPs were also observed to be better than the uncoated AgNPs in terms of size distribution, morphology, and stability.

Furthermore, even though PEG-coated AgNPs (from biological synthesis) demonstrated lower antimicrobial activities than their uncoated counterparts (i.e., less than 31% reduction), the amalgamation may be useful in drug delivery systems. Hence, the present study highlighted the interrelations of the synthesis method, PEG coating, physico-chemical characteristics, and biological activities (antimicrobial) of AgNPs.

## Figures and Tables

**Table 1:** Main absorption peaks and vibrational assignments with functional groups interpretation of the uncoated and PEG-coated AgNPs produced from chemical and biological synthesis [9,15,26,27].

Wavenumber (cm^−1^)				Vibrational Assignment	Functional Group
Chemical Synthesis		Biological Synthesis			
Uncoated AgNPs	PEG-Coated AgNPs	Uncoated AgNPs	PEG-Coated AgNPs		
3268	3359	3294	3270	O–H stretching	Hydroxyl
-	2921	-	2920	C–H stretching	Carboxylic acid
1635	1638	1635	1635	C=O stretching, N–H stretching	Amide Ⅰ
-	1463	-	1469	C–H bending	Aromatic compounds
-	1351	-	1351	O–C–H	Alcohols
-	1252	-	1298	C–C stretch	Ketones
-	1081	-	1083	C–C=O, C–O–P	Hydroxyl from saccharides
-	946	-	945	C–H bend	Alkenes

**Table 2:** The polydispersity index and zeta potential measurements of AgNPs determined using Zetasizer Nano.

Parameter	Chemical Synthesis		Biological Synthesis	
	AgNPs	PEG-Coated AgNPs	AgNPs	PEG-Coated AgNPs
Polydispersity index (PdI)	0.514	0.476	0.571	0.534
Zeta potential (mV)	−32.3	−36.4	29.8	−30.6

**Table 3:** Inhibitory activities of AgNPs from chemical and biological syntheses against multidrug-resistant bacterial strains.

Samples		Zone of Inhibition (mm) for 100 µL			
		Gram Positive Bacteria		Gram Negative Bacteria	
		*Staphylococcus aureus*	*Staphylococcus epidermidis*	*Escherichia coli*	*Salmonella* sp.
Ampicillin (control)		29.75 ± 1.28	41.50 ± 1.41	17.50 ± 2.98	69.2 ± 12.30
Chemically Synthesised AgNPs	Uncoated	0.00	0.00	0.00	0.00
	PEG-Coated	0.00	0.00	0.00	0.00
Biologically Synthesised AgNPs	Uncoated	23.00 ± 1.41	20.00 ± 0.00	13.00 ± 1.41	26.00 ± 0.00
	PEG-coated	16.00 ± 0.00	14.00 ± 0.00	12.00 ± 0.00	18.00 ± 0.00

All values represented are the average of three replicates conducted experiment.
